# Why trees grow at night

**DOI:** 10.1111/nph.17552

**Published:** 2021-07-07

**Authors:** Roman Zweifel, Frank Sterck, Sabine Braun, Nina Buchmann, Werner Eugster, Arthur Gessler, Matthias Häni, Richard L. Peters, Lorenz Walthert, Micah Wilhelm, Kasia Ziemińska, Sophia Etzold

**Affiliations:** ^1^ Swiss Federal Institute for Forest, Snow and Landscape Research WSL Birmensdorf 8903 Switzerland; ^2^ Forest Ecology and Management Group Wageningen University Wageningen 6708 PB the Netherlands; ^3^ Institute for Applied Plant Biology Witterswil 4108 Switzerland; ^4^ Department of Environmental Systems Science Institute of Agricultural Sciences ETH Zurich Zurich 8092 Switzerland; ^5^ Laboratory of Plant Ecology Ghent University Ghent 9000 Belgium; ^6^ Department of Plant Ecology and Evolution Uppsala University Uppsala SE‐751 05 Sweden

**Keywords:** cell turgor threshold, climate change, day–night radial stem growth, dendrometer, ecophysiology, photoperiod, wood and bark formation, xylogenesis

## Abstract

- The timing of diel stem growth of mature forest trees is still largely unknown, as empirical data with high temporal resolution have not been available so far. Consequently, the effects of day–night conditions on tree growth remained uncertain.
- Here we present the first comprehensive field study of hourly‐resolved radial stem growth of seven temperate tree species, based on 57 million underlying data points over a period of up to 8 yr.
- We show that trees grow mainly at night, with a peak after midnight, when the vapour pressure deficit (VPD) is among the lowest. A high VPD strictly limits radial stem growth and allows little growth during daylight hours, except in the early morning. Surprisingly, trees also grow in moderately dry soil when the VPD is low. Species‐specific differences in diel growth dynamics show that species able to grow earlier during the night are associated with the highest number of hours with growth per year and the largest annual growth increment.
- We conclude that species with the ability to overcome daily water deficits faster have greater growth potential. Furthermore, we conclude that growth is more sensitive than carbon uptake to dry air, as growth stops before stomata are known to close.

## Introduction

Species‐specific responses of tree growth to environmental conditions are crucial for understanding forest dynamics in a world with a rapidly changing climate (Babst *et␣al*., 2019; Bastos *et␣al*., 2020; McDowell *et␣al*., 2020). However, knowledge about the physiological and environmental drivers of tree growth is still limited by the temporal resolution of dendrochronological methods. Such data are often coarse and are obtained typically with an annual resolution of the underlying tree ring samples (Schweingruber, 1996; Babst *et␣al*., 2018) or, in less frequent cases, small wood samples obtained at (bi‐)weekly intervals (Zweifel *et␣al*., 2006; Delpierre *et␣al*., 2016; Huang *et␣al*., 2020; Peters *et␣al*., 2021). Daily or even hourly‐resolved growth responses of mature forest trees have been rarely recorded (Schurr *et␣al*., 2006), most data covers only short periods and were monitored in single stands (Ziaco & Biondi, 2018; Knüsel *et␣al*., 2019; Güney *et␣al*., 2020).

However, there is an urgent need for growth information with sub‐daily resolution, since the average conditions over a week or a year might affect growth differently than within a specific hour. Growth is not a continuous, linear process but is strongly determined by thresholds of plant water potentials as has been shown for several tree species (Lazzarin *et␣al*., 2019; Cabon *et␣al*., 2020; Peters *et␣al*., 2021). These water potentials vary strongly with the time of day and climate change effects may therefore greatly differ between day and night.

Despite the current lack of hourly‐resolved growth data, the theoretical background to explain the mechanisms of cell division and cell expansion in a tree stem is well established (Woodruff & Meinzer, 2011). At the core of the proposed mechanism is the soil–plant–atmosphere continuum including the cohesion‐tension theory (Dixon & Joly, 1894), which connects soil water dynamics with tree hydraulics and atmospheric water demand and determines the water potential in the cambium where the new wood and bark cells are formed (Woodruff & Meinzer, 2011). Vapour pressure deficit (VPD) and soil water potential (SWP) become the key drivers of the water potential gradient that determines the water flow through the tree (Steppe *et␣al*., 2006; Novick *et␣al*., 2016; Carminati & Javaux, 2020). These two variables reflect the water potentials in the air and soil as well as integrate over other environmental variables, for example, temperature and relative humidity.

The essentials about the physiological relationship between tree water relations and growth dates back to the 1960s, when Lockhart (1965) described how a turgor threshold of the meristem must be crossed before cell expansion and cell division is promoted. Many later studies supported this theory (Steppe *et␣al*., 2006; Muller *et␣al*., 2011; Lazzarin *et␣al*., 2019; Cabon *et␣al*., 2020; Peters *et␣al*., 2021). Lockhart's theory implicitly considers growth not only as a function of the carbon source but also of the water conditions since varying environmental conditions may independently determine carbon uptake and turgor pressure (Fatichi *et␣al*., 2014).

**Fig. 1:**
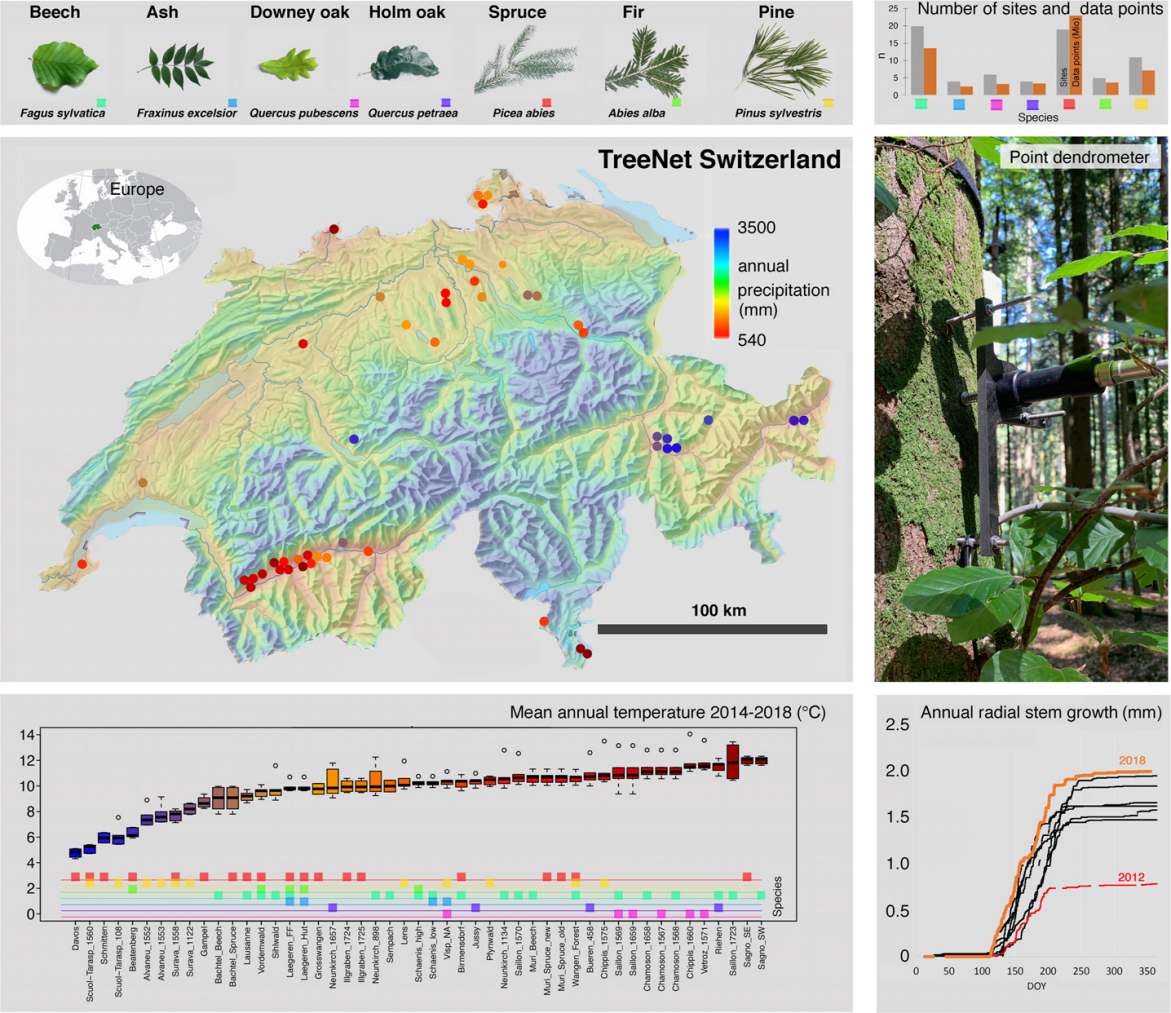
Location and characteristics of the 50 TreeNet sites in Switzerland (www.treenet.info). Seven tree species including data of 170 individually measured trees (Supporting Information Table S1) were investigated with a total of 57 million underlying data points at a 10‐min resolution (species‐specific sums, panel top right) that were aggregated to hourly averages for analyses. The background colour of the map indicates the average annual precipitation. The site dots are colour‐coded according to the mean annual temperature shown in the boxplot panel (lower left). The boxplots indicate the median (horizontal bar) mean annual temperature, with the boxes representing the 25–75% quartile range and the whiskers the lowest and highest value within 1.5× the interquartile (IQR, 75–25%) range. Circles define outliers beyond the 1.5×IQR. Mean annual temperature and precipitation cover a large gradient from 4.5 to 11.9°C and from 540 to 1700 mm, respectively. The photograph shows a point dendrometer mounted on a beech tree and the panel below gives the corresponding growth performance over 8 yr, highlighting the years with the lowest and the largest stem radial growth.

The turgor threshold theory also enabled the development of a method to extract hourly stem radial growth data from continuous, high‐precision dendrometer measurements (Fig. 1; Supporting Information Fig.␣S1). Dendrometer data consist of radial increment due to growth and tree water related shrinkage and expansion of mainly the bark, which need to be separated prior to analysing stem growth. A recent study (Zweifel *et␣al*., 2016) presented a separation method (zero‐growth approach) based on empirical and theoretical evidence that radial stem growth is suppressed when tree stems are shrinking due to the transpiration‐induced lowering of the turgor pressure. In short, growth is equivalent to an incremental increase in stem radius when the measured radius is larger than it was at any point in the past. The authors estimated that > 95% of the growth could be attributed to the correct hour, making for a very robust and credible, but not perfect, separation approach.

There remain uncertainties with potential hygroscopic effects of the bark of some tree species when the stem surface is moistened by rain (Oberhuber *et␣al*., 2020). Further, the zero‐growth approach does not consider the still largely unknown bark degradation processes (Gricar *et␣al*., 2015; Güney *et␣al*., 2020), especially after frost in the winter period (Zweifel & Häsler, 2000; Charrier *et␣al*., 2017). Uncertainties also remain due to technical issues, for example, the electronic and mechanical temperature behaviour of dendrometers, the way the sensor is anchored in the tree, or the way the raw data is cleaned from outliers and shifts (Haeni *et␣al*., 2020). As there is so far no alternative method capable of measuring radial stem growth with a similarly high resolution on mature forest trees, the zero‐growth approach lacks an ultimate quality control. The application of another recently developed empirical approach to separate growth from dendrometer data (Mencuccini *et␣al*., 2017) did not offer an alternative, as the remaining uncertainties are the same and the approach additionally needs sap flow data not available in this infrastructure.

Meanwhile, the zero‐growth approach has become widely accepted and applied (Dietrich & Kahmen, 2019; Schafer *et␣al*., 2019; Eitel *et␣al*., 2020; Güney *et␣al*., 2020; Lamacque *et␣al*., 2020; Pappas *et␣al*., 2020; Sellier & Segura, 2020). Accordingly, growth is defined as the radial stem increase above a previously reached stem radial maximum (Fig. S1). All stem size changes below this dynamically increasing maximum were attributed to the water conditions of the trees and removed from the growth data. Growth is understood as irreversible stem radial increment, including new bark and wood cells and neglecting any cell maturation processes (e.g. lignification) (Cuny *et␣al*., 2015; Rathgeber *et␣al*., 2016).

Here, we present the first comprehensive study of hourly‐resolved stem growth data from a large, technically homogenous network of 170 trees at 50 sites, continuously measuring stem radius and air and soil conditions (Fig. 1). The key hypotheses of this study address the sensitivity of growth to VPD and SWP as well as the dependence of stem growth on the time of day:

1. We expect diel stem growth to increase overnight and decrease during the day because transpiration reduces water potential and turgor pressure in the cambium, thus inhibiting cell division and cell expansion.
2. We expect stem growth to be favoured under humid conditions and thus improved under conditions of low VPD and high SWP. Furthermore, we expect that stem growth always responds similarly to environmental conditions, regardless of the time of day.
3. We expect stem growth to be species‐specific as species are known to have different sensitivities to VPD and SWP (e.g. stomatal regulation) and other factors (e.g. circadian rhythm).

## Materials and Methods

### Sites and setup

The 50 sites studied are part of TreeNet (www.treenet.info), a network where stem radius changes of trees are measured continuously using high‐precision point dendrometers, in parallel with environmental information of air (VPD) and soil (SWP) in Swiss forests since 2011 (Fig. 1). Most forests were managed sustainably since 1876 and are either deciduous, evergreen or mixed.

Four angiosperms (*Fagus sylvatica*, *Fraxinus excelsior*, *Quercus pubescens*, *Quercus petrea*) and three gymnosperms (*Picea abies*, *Pinus sylvestris*, *Abies alba*) were analysed between 2011 and 2018 (Fig. 1). All of them fulfilled the minimum criteria of appearing at > 3 sites with > 2 yr of data per time series and a minimum of two individuals per site. Further, only vital, mature and dominant trees that grew more than 100 µm yr^−1^ were included. The total number of trees per species ranged from 7 to 62. All analyses were based on hourly‐resolved data. Further characteristics of species and sites are listed in Supporting Information Table S1.

### Environmental conditions

Air temperature (TEMP) and relative humidity (RelH) was obtained either from weather stations located at the sites (sources: treenet.info and lwf.ch) or from nearby (mean distance: 8.3 km, maximum: 15.4 km) MeteoSwiss stations (meteoswiss.admin.ch). VPD was calculated from TEMP and RelH.

SWP (MPS‐2/MPS‐6; Decagon Devices, Pullman, WA, USA) was measured at 10–20 cm soil depth at each of the 50 sites (treenet.info) and corrected for soil temperature (Walthert & Schleppi, 2018). These topsoil water potentials (SWP values) were cross‐checked with SWP measurements over 2 m profiles at five sites (Fig. S2).

### Dendrometer measurements

Point dendrometers (ZN11‐T‐IP and ZN11‐T‐WP, Natkon, Oetwil am See, Switzerland, Fig. 1) were mounted on the stem at breast height. The dendrometers consisted of a carbon‐fibre frame with three stainless steel threaded rods which served as anchors in the (heart) wood of the tree stem. A metal sensing pin was gently pressed against the surface of the bark by a spring. The electronic transducer detected changes in the position of the pin and thus stem radius changes measured over the bark. The outermost dead layer of bark under the pin was carefully removed to minimize the effect of hygroscopic swelling. Special care was taken to avoid damaging the living bark underneath.

The dendrometer data were recorded and transmitted with DecentLab logging devices (DecentLab GmbH, Dübendorf, Switzerland) at 10 min intervals or better. The logging resolution was < 1 µm and the temperature sensitivity of the applied dendrometers (including all components and logger) was < 0.3 µm °C^−1^ and was thus negligible.

### Stem radius fluctuations and growth calculations

Dendrometer data combine two major processes of the tree stem physiology: irreversible growth and reversible swelling and shrinkage (Fig. S1). Here we use the so called ‘zero growth’ concept for stem radius partitioning into periods with and without growth, which assumes no growth during periods of stem shrinkage and is explained in detail elsewhere (Zweifel *et␣al*., 2016). Following this concept, the accumulative growth (GRO) increases in periods when the stem radius exceeds its previous maximum (GRO > 0). During the remaining time, stems either shrink or expand below this maximum and the deviation to the maximum is called tree water deficit (TWD > 0), or the stem radius exactly meets its previous maximum (TWD = 0, GRO = 0).

### Stem growth period and growth hours

The stem growth period was defined as the period of the year when radial increments were detected and was determined separately for each site and species (based on the average of all trees per species and site). The first and last 5% of the stem growth period were excluded to avoid outliers influencing the timing of the growth periods. Hours with growth were flagged as ‘growth hours’, summed up for each individual tree and year (‘growth hours per year’), and further aggregated to species‐specific median growth hours per year.

### Data treatment and statistical methods

All analyses and plots were made with R statistical software (R_Core_Team, 2019). The raw dendrometer data of each tree (10‐min resolution) over the full length of the time series were quality checked and processed with the R‐package treenetproc (Haeni *et␣al*., 2020). For analyses or calculations that were based on annual values we included annual time series that covered > 90% of the stem growth period only.

Statistical analyses of diel, hourly resolved data were based on aggregated data considering the nested data structure. The aggregation process underwent three steps. First, the daily growth pattern for each tree individual was calculated as the relative contribution of a given hour to the total annual growth (in percentage; the 24 h of a day equal to 100%) in order to normalize the highly varying absolute annual growth between individual trees and to aggregate the data over several years. Second, these relative growth numbers were averaged per site and species. And thirdly, the data were pooled into (1) a growth response of all species and (2) aggregated into a species‐specific response pattern. The VPD and SWP data were treated in the same way. All steps included medians with 25% and 75% quantiles. Based on these aggregated data sets, growth probability (%) was quantified as the number of hours with growth relative to the total number of hours in the stem growth phase. Hourly growth rates in absolute units (µm h^−1^) were calculated accordingly, but not normalized for variations in annual increments.

Multiple linear regression models were applied to test the relationships between VPD and SWP and the daily growth cycle at hourly resolution of these aggregated data sets. Contour diagrams of hourly growth in relation to VPD and SWP were made with the R‐packages gridextra (Auguie & Antonov, 2017) and reshape2 (Wickham, 2020), and are based on a local polynomial regression (loess) function that interpolates growth in relation to time of day and environmental conditions. This interpolation model covered on average 70% of the underlying data variation of individual species. Uncertainty analyses were performed using a bootstrapping procedure in which a selection of data was randomly resampled 1000 times and the coefficient of variation was calculated over these data.

## Results

### Stem growth at night

**Fig. 2:**
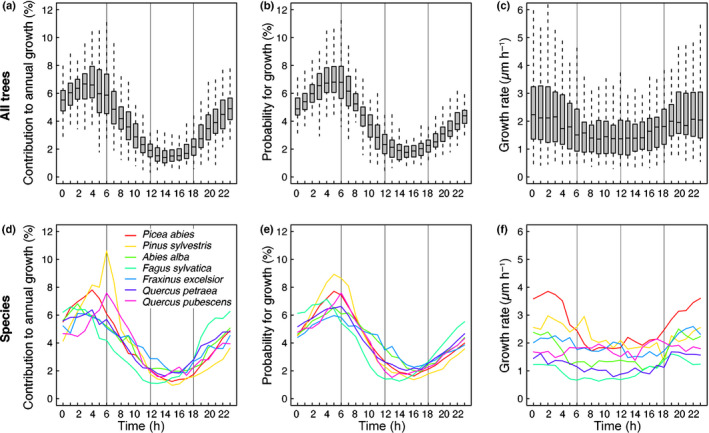
Diel growth of seven temperate tree species. Boxplots show median, 25 and 75 percentiles and the SD of the pooled data for all species (a–c). Lines show the medians of each species (d–f). The relative contribution of a specific hour to the annual growth is shown in (a, d). The probability for growth quantified as number of hours with growth relative to the total number of hours in the stem growth period is shown in (b, e) and the median hourly growth rates of hours with growth are shown in (c, f).

Over a diel cycle, all tree species grew mainly at night, with the highest contribution to total stem growth just before dawn and the lowest contribution to growth during midday and in the afternoon (Fig. 2a), which supported hypothesis (1). The probability for growth (Fig. 2b), quantified as number of hours with growth relative to the total number of hours in the stem growth period, largely explained the diel growth pattern (72% in a multiple regression model), while hourly growth rates (Fig. 2c) remained relatively stable over the diel cycle and explained much less (26%). In addition to the general pattern for all species, the hourly contribution to annual growth (Fig. 2d), growth probability (Fig. 2e) and growth rate (Fig. 2f) showed species‐specific features, for example, the timing of maximum and minimum stem growth as listed in Table 1. For simplicity, from here on we use the term ‘stem growth’ to refer to the hourly, relative contribution of growth to annual growth (Fig. 2a,d) and ‘growth rate’ for the stem growth rate per hour (Fig. 2c,f).

**Table 1:** Species‐specific features of diel growth in relation to annual growth.

	GRO.hr (µm h^−1^)	Time.GRO.max (time of day)	Time.GRO.min (time of day)	*R*^2^_VPD_ (adj *R* ^2^)	h.GRO.yr (h)	GRO.yr (µm)
*Abies alba*	1.64	2	16	0.60	746	1817
*Fagus sylvatica*	0.94	1	13	0.81	606	1159
*Fraxinus excelsior*	2.02	2	17	0.72	572	1439
*Picea abies*	2.50	4	15	0.65	468	1507
*Pinus sylvestris*	2.29	6	15	0.50	301	795
*Quercus petrea*	1.14	4	14	0.94	540	1044
*Quercus pubescens*	1.69	6	14	0.68	280	656

Listed are the median hourly growth rates calculated for hours with growth (GRO.hr), time of day when the diel contribution to annual growth was at its maximum (Time.GRO.max) and at its minimum (Time.GRO.min). Adjusted *R* ^2^ (*R* ^2^_VPD_) are listed for the linear regression of the two time series. h.GRO.yr represents the median total number of hours with growth per year, while GRO.yr represents the median radial increment per year.

### Stem growth in relation to dryness in air and soil

**Fig. 3:**
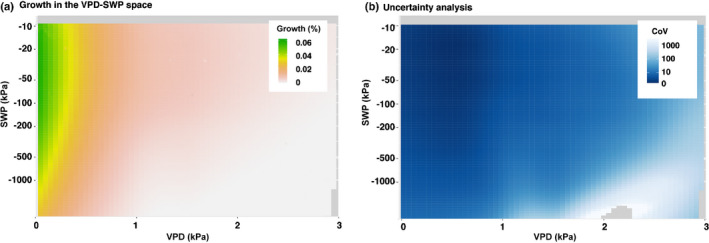
Hourly‐resolved, radial stem growth and the corresponding uncertainty analysis in the measured space of vapour pressure deficit (VPD) and soil water potential (SWP) across all species. (a) Growth was quantified as the relative hourly contribution to the total annual growth (per grid element) and ranged from white (no growth, 0%), over red (marginal growth, 0.02%) to dark green (high growth, 0.06%). (b) The coefficient of variation (CoV) of the uncertainty analysis indicated the robustness of the results between very good (values < 10, dark blue), good (10 to < 20), and satisfactory (20–50), to poor (> 50, light blue to white). The interpolation output of the contour diagram was restricted to the effectively measured range of environmental variables. Grey areas indicate no data. Species‐specific growth responses can be found in the Supporting Information Fig. S3.

In general, main growth was limited to a VPD < 0.4 kPa and a SWP > −900 kPa corresponding to the greenish area in Fig. 3(a), covering more than 75% of total growth. The result was found to be robust in terms of a low uncertainty for growth within the relevant VPD and SWP ranges (Fig. 3b). The species‐specific VPD thresholds for main growth ranged within a narrow band of values for all species, whereas the SWP threshold considerably varied across species (Fig. S3). *Quercus pubescens* grew at the lowest SWP by far, whereas *Abies alba* covered moist soil conditions only (Table S2). Further, the available SWP for all sites (measured at 10–20 cm soil depth) largely represented the temporal dynamics of five available soil profiles of 2 m depth. However, the lower boundary for main growth was shifted by about 200–300 kPa upwards between the data used in this study (Fig. 3) and the data from the 2 m soil profile (Fig. S2). The median SWP were hardly differing (Table S2).

VPD and SWP together explained on average 81% of the diel growth variation of all species in a multiple regression model (Table S3). A model that included all available environmental variables (relative humidity, air temperature, net radiation, SWP and the respective pairwise interactions) explained only slightly more, namely 88% (Table S4).

The explanatory power of SWP for diel growth varied considerably among species (Table S3) between 0% (*Quercus petraea*) and 27% (*Abies alba*), and was significantly higher for the conifers (20%) than for the deciduous species (5%). Growth decreased for a given VPD with decreasing SWP in some species (*Picea abies*, *Abies alba*, *Quercus petraea*), while interestingly it increased in other species up to a certain SWP (−30 to −150 kPa) and only decreased thereafter (Fig. S3). Or in other words, not all species had the greatest growth under the wettest soil conditions (measured in 10–20 cm).

### Diel dynamics of stem growth in relation to vapour pressure deficit and soil water potential

**Fig. 4:**
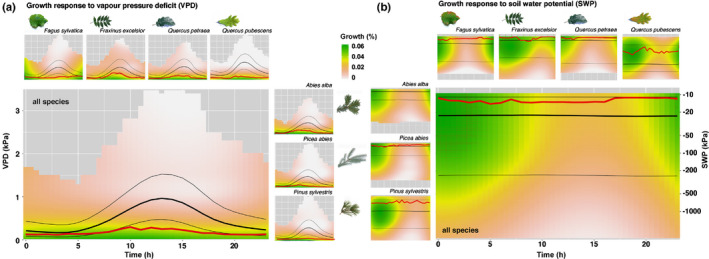
Diel radial stem growth in relation to (a) vapour pressure deficit (VPD) and (b) soil water potential (SWP). Growth is colour‐coded and ranges from white (no growth, 0%), through red (little growth, 0.02%) to dark green (high growth, 0.06%). The main panel shows the general response over all species whereas the small panels show the species‐specific responses (same axis). The bold black line indicates the median VPD and SWP conditions in the stem growth period (thin black lines indicate 25%‐ and 75%‐quantiles). By contrast, the bold red line shows the same but for hours with growth only. A plot with the underlying measurement data can be found in Supporting Information Fig. S4. Growth is quantified as the relative growth contribution to the total annual growth and is based on aggregated data sets considering the nested design of trees and species within sites. The interpolation output of the contour diagram was restricted to the effectively measured range of VPD and SWP. Grey areas indicate no data.

The general relationship between the diel growth and the environmental variables VPD and SWP appeared robust across all species (main panels, Fig. 4), resembled the patterns for individual species (small panels, Fig. 4), and was largely independent of the time of the season (Fig. S5). Diel growth responded more consistently to VPD, while the growth response to SWP was strongly depending on the time of day. A particular SWP resulted in higher or lower growth depending on the actual time of day, with nocturnal conditions allowing growth over a much larger SWP range compared to daylight hours.

In all species, the median VPD for hours with growth (bold red lines, Fig. 4) was consistently lower than the median VPD of all hours in the entire growth period (bold black line, Fig. 4). Especially during daylight hours, the difference between the two lines increased, while the same deviation for SWP was less dependent on the time of day. However, both the soil and the air were generally drier than required for growth, allowing only a limited number of hours with growth throughout the entire stem growth period. *Quercus pubescens* and *Pinus sylvestris* had the fewest hours with growth (*c.* 300), while *Fagus sylvatica* and *Abies alba* had more than double (Table 1).

### Timing of peak growth as indicator for annual growth

Despite the robust 24‐h growth pattern across all species and sites, there were clear species‐specific differences where the growth of some species peaked earlier at night than of others (Fig. 2d; Table 1). *Fagus sylvatica* started to increase (13:00 h) and reached the peak (01:00 h) of its median diel growth earlier than all other species, which had growth peaks between 02:00 h and 06:00 h in the early morning and growth minima during midday and early afternoon, between 14:00 h and 16:00 h (Table 1). Of all species, *Pinus sylvestris* showed the most pronounced growth peak (highest amplitude, Fig. 2d), followed by *Picea abies* and *Quercus pubescens*, indicating that growth was more restricted to these specific hours. By contrast, *Quercus petraea* and especially *Fraxinus excelsior* had the least pronounced growth maxima and minima, indicating that growth was more evenly distributed over the 24 h than in other species.

**Fig. 5:**
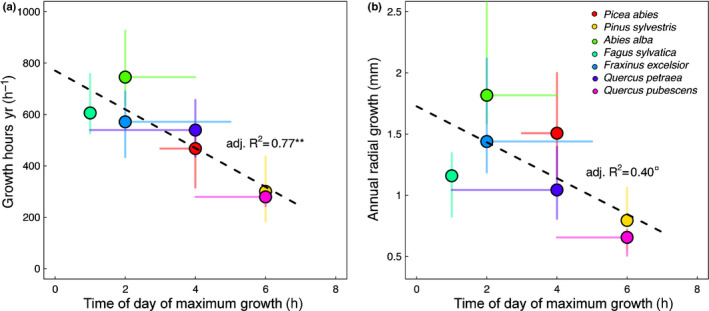
Time of day of maximum growth in relation to (a) the number of hours with growth and (b) the radial stem increment per year. Shown are the medians (circles) and the range between the 25%‐ and 75%‐quantiles (lines) of each species (colour‐coded). Dashed lines show the linear regression. **, *P* < 0.01; ^□^, *P* < 0.1.

Strikingly, the species‐specific temporal growth peaks were very closely negatively related with the annual, species‐specific number of hours with growth (adj.*R* ^2^ = 0.77; Fig. 5a; Table 1) and also with the annual radial stem growth (adj.*R* ^2^ = 0.4; Fig. 5b). Species with their growth peak earlier in the night generally had more hours with growth per year (*Fagus sylvatica*, *Abies alba*, *Fraxinus excelsior*) and also generally grew more than those species with a growth maximum later in the night (*Pinus sylvestris*, *Quercus pubescens*).

## Discussion

### Consistent diel growth patterns across species

We found that the trees grew mainly at night, with species‐specific growth peaks between 01:00 h and 06:00 h (Figs 4, 5; Table 1), when the trees are presumed to be best replenished with water and thus likely to provide the best conditions to exceed the turgor threshold for cell division and cell expansion (Schurr *et␣al*., 2006; Cabon *et␣al*., 2020). This timing of growth was consistent with the observation that it takes several hours after nightfall for stems to replenish and to compensate for the tree water deficit caused by the imbalance between transpiration and water uptake during daylight hours. It is this water deficit, which must be compensated for before growth processes are proposed to become likely (Zweifel *et␣al*., 2016). Stem growth also occurred during daylight hours (Fig. 2), but with a consistently lower relative contribution to annual growth. Overall, these results are consistent with our hypotheses, which emphasize the importance of tree water relations and their determination by atmospheric and soil dryness, in line with the turgor pressure threshold concept for growth (Lockhart, 1965; Peters *et␣al*., 2021).

### Different effects of vapour pressure deficit and soil water potential on growth

Radial stem growth was associated with high mean SWP (−6 to −65 kPa) and low mean VPD (0.11–0.24 kPa, Table S2), emphasizing the commonly known negative influence of dry air and soil on growth processes (Zweifel *et␣al*., 2006; Pappas *et␣al*., 2020). However, these daily mean values did not account for diel dynamics and they did not reflect how differently SWP and VPD were generally related to stem growth at an hourly resolution (Figs 3, 4). VPD consistently limited the main growth (> 75% of annual growth) to a range < 0.4 kPa (Fig. 3), largely independent of species (Fig. S3) and time of day (Fig. 4), while SWP showed a much wider and more species‐specific range of main growth (> −900 kPa). Especially at night (Fig. 4), stem growth consistently occurred even at lowered SWP, suggesting that growth is possible under moderate soil dryness if VPD is low enough at the same time. The more linear relationship between VPD and stem growth (over 24 h) than that between SWP and growth (Fig. 4) also underlines recent findings that VPD increasingly limits tree growth due to an increasingly warmer climate (Novick *et␣al*., 2016; Grossiord *et␣al*., 2020; Peters *et␣al*., 2021).

Increased VPD also restricted stem growth under wet soil conditions, because high atmospheric water demand on a sunny day increased transpiration faster than root water uptake, leading to tree water deficit (Hinckley & Bruckerhoff, 1975; Zweifel *et␣al*., 2005) and presumably reduced turgor pressure below the growth threshold in the cell‐dividing tissue, i.e. the stem cambium (Steppe *et␣al*., 2006; Cabon *et␣al*., 2020). Because the observed VPD threshold for main growth was so low and VPD generally increases so rapidly with daylight, growth inhibition occurred on any sunny day (Fig. 4). Growth in the afternoon was only observed when VPD remained low, that is, on rainy or cloudy days. This was sometimes even the case when little rain fell during a period with dry soils (Fig. S6). However, the additional meteoric water was not sufficient to moisten the soil to the depth where the soil sensors were installed (10–20 cm), but may have increased water uptake by the topmost roots near the soil surface. In addition, small rain events moisten the surface of a tree, reduce transpiration and can rehydrate the plant via water uptake through the leaves (Goldsmith *et␣al*., 2017) and thus have the potential to increase negative water potentials and could also raise turgor pressure above the growth threshold from above.

The importance of humid air for the physiological performance of a tree has also been demonstrated for the redwood tree giants (Simonin *et␣al*., 2009). Our results suggest the importance of small rain events not only for these giants, but most likely also for the growth of temperate tree species, particularly in an increasingly drier environment (McDowell *et␣al*., 2020), as also recently suggested by Dietrich & Kahmen (2019). Low VPD is most likely not directly responsible for growth, but it seems to be a strong indicator for leaf surface wetness, which induces the necessary relaxation of low water potentials. However, SWPs that were too low (Fig. 3) did not allow for growth either. We assume that under such conditions the tree's growth physiology cannot be sufficiently stimulated by atmospheric moisture alone. Overall, our results suggest that under moderately dry soil conditions, information on air humidity is essential to accurately estimate stem growth.

### Stem growth is more drought‐sensitive than carbon assimilation

A VPD > 0.4 kPa strongly reduced stem growth and for a VPD > 1 kPa it was only marginal (Figs 3, 4). The low VPD threshold for growth thus seems to be in marked contrast to the initial stomatal response to VPD, which is known to start at a VPD of > 0.5 kPa for probably all tree species (Oren *et␣al*., 1999; Grossiord *et␣al*., 2020) and could reach values of up to > 2 kPa in more drought‐adapted species, for example, *Quercus* (Gil‐Pelegrin *et␣al*., 2017). Moreover, above the VPD threshold for the initial response, the entirety of the stomata do not suddenly close completely but gradually limit CO_2_ uptake as VPD continues to increase (Hetherington & Woodward, 2003). This implies that the suppression of carbon assimilation by fully closed stomata is, therefore, associated with an even higher VPD that significantly exceeds that for potential stem growth.

We interpret the two different sensitivities of growth and stomata to VPD as indicating that growth processes are generally more sensitive to atmospheric drought than carbon assimilation, as suggested by recent studies (Muller *et␣al*., 2011; Korner, 2015), but with the novel aspect that stem growth depends mainly on nocturnal conditions, while assimilation depends on daytime conditions. As VPD rapidly increases in the morning, carbon assimilation remains active much longer than growth during daylight hours. This makes physiological sense from three different points of view: First, carbon assimilation of trees is only possible during sunlight hours and would become very inefficient if the stomata were already starting to close with the rising sun. Second, growth does not necessarily have to take place in daylight, as our results clearly show (Figs 2, 4). And third, an organism that shuts down its carbon sink before its carbon source could potentially reduce the risk of resource depletion during prolonged drought stress (Rowland *et␣al*., 2015). However, stem growth is not the only carbon sink in a tree, and it is likely that other organs are sinks at different times and could therefore alter the carbon reserve dynamics independently.

Since the two processes of growth and carbon assimilation are so clearly associated with different VPD ranges, they are also temporally separated into daylight hours for carbon assimilation and night hours for growth. Our results thus imply a temporal decoupling of the processes of carbon source and sink dynamics for growth even within the 24‐h cycle, as recently proposed for the seasonal carbon balance (Korner, 2015; Gharun *et␣al*., 2020). Thus, growth seems not primarily source‐controlled by the actual carbon uptake, but rather a process that is highly dependent on tree water relations and the current environmental conditions, i.e. soil moisture and air humidity.

### Species‐specific diel timing of growth

Despite the generality of the daily growth trends, the seven species studied consistently differed in the timing of the growth peaks (Table 1; Fig. 5), and it was striking that the earlier a nocturnal growth peak of a species was reached, the higher the number of hours with growth per year and the annual radial growth of the stem were (Fig. 5).

Interestingly, we did not find a consistent grouping of species in Fig. 5 with respect to wood types (gymnosperms, angiosperms), wood anatomy (ring‐porous, diffuse‐porous, tracheids) or foliage type (evergreen needles, deciduous leaves). However, the ranking of species along the linear regression line between time of day of maximum growth and the annual number of hours with growth was at least partly explainable by the respective SWP and VPD ranges for growth (Table S2). The drier the average site conditions where a species occurred, the fewer hours with growth were measured, which is consistent with the generally known limitation of growth by drought (McDowell & Sevanto, 2010; Korner, 2015; Pappas *et␣al*., 2020). This was especially true for the two most extreme species in our study, *Abies alba* (growing mostly under humid conditions) and *Quercus pubescens* (growing under driest conditions). Though, this was not consistently true for all species between these two extremes (Tables 1, S2), which might also be due to averaging the wide range of conditions that some of the species covered (e.g. *Pinus sylvestris*, Fig. 1).

The very close linear relationship between the timing of the diel growth peak and the annual number of hours with growth (adj.*R* ^2^ = 0.77) suggests a general mechanistic reason governing it. We speculate that those species that are able to grow earlier at night (Fig. 2) generally have more hours available under suitable conditions per night and therefore have a better chance of accumulating more hours with growth over the season. However, the results also showed that the species with an earlier growth peak (*Fagus sylvatica*, *Abies alba*, *Fraxinus excelsior*) also reduced their growth earlier (Fig. 2) and therefore did not take full advantage of the generally moistest conditions around dawn. By contrast, species with a late growth peak (*Pinus sylvestris*, *Quercus pubescens*) may not have had enough time to grow long enough due to the environmental constraints, resulting in pronounced growth peaks around dawn (Fig. 2), a reduced number of growing hours per night and per growing season, and thus reduced absolute annual stem growth (Fig. 5).

### What alters the species‐specific nocturnal growth peaks?

The generally drier conditions under which some of the species grew could be part of the answer. However, we speculate on other reasons as well. First, the species‐specific morphological differences, for example, bark thickness or water transport capacity of the wood (Steppe *et␣al*., 2006) could play a role. *Fagus sylvatica*, as the species with the earliest growth peak, has by far the thinnest bark tissue of the species studied (Ilek & Kucza, 2014) and therefore may need less water and time to replenish its shrunken tissue. Further, an efficient water transport system such as the ring‐porous wood of *Fraxinus excelsior* could help to accelerate the replenishment of the tree's water deficit (Brinkmann *et␣al*., 2016; Klesse *et␣al*., 2020) and thus induce a faster increase in cambium turgor after nightfall. Second, the increase of turgor pressure could additionally be enhanced by osmoregulation, a process actively altering a cambium cell’s turgor even during periods when water potentials in the xylem are low due to increased transpiration, as has recently been shown for several tree species (Zweifel *et␣al*., 2014; Barraclough *et␣al*., 2019; Lazzarin *et␣al*., 2019). And third, the circadian rhythm has been shown to play an important role in explaining species‐specific phenological traits, for example, bud burst or the molecular regulation of annual growth (Singh *et␣al*., 2017; de Dios & Gessler, 2018; Huang *et␣al*., 2020). *Fagus sylvatica* is known to follow the circadian rhythm (de Dios *et␣al*., 2015) more closely than other species. We, therefore, speculate that there might also be an influence of the circadian clockwork on diel growth. Our assumption is based on the unique, temporally irregular pattern of the growth response to VPD of *Fagus sylvatica* (Fig. 4). *Fagus* is the only species where the otherwise constant relationship between VPD and growth disappeared during the night. Such a pattern suggests a decoupling of growth from VPD and would for example be expected if a species closed its stomata completely regardless of environmental conditions, for example, initiated by a circadian rhythm. Such a mechanism also has the potential to explain the unusual diel growth pattern of *Fagus sylvatica* and possibly other species.

### Potential limitations of the results

Any result is only as good as its underlying data quality and premises. This work relies on technically correct measurements of radial stem size changes in micrometre resolution, consistent and reproducible handling of the partially erroneous dendrometer raw data (Haeni *et␣al*., 2020) and on the zero‐growth approach that separates the dendrometer data into irreversible radial growth due to new cells and reversible swelling and shrinking due to water relations in the tree (Zweifel *et␣al*., 2016).

The homogeneous technical design of the network (www.treenet.info) with the use of only one type of high‐precision point dendrometers, the uniform hardware for recording the data, the automatic data transfer to a central database and the automated routine for uniform cleaning and quality checking of the raw data helped to optimize the reliability of the data. However, the remaining technical uncertainties due to undetected data outliers despite the automated cleaning procedure and manual data checking, or the remaining temperature sensitivity of the measuring instruments despite a setup developed to minimize this effect, can never be completely avoided. In this work, it can be assumed that such effects are levelled out by the aggregation steps of the huge data set, or affect all measurements in the same systematic way and are thus of little relevance to the results presented. Also not to be completely excluded are potential artefacts due to episodic hygroscopic swelling of the bark when the stem surface is wetted by rain (Oberhuber *et␣al*., 2020). However, this potential effect was minimized by using point dendrometers (not band dendrometers) and removing the dead outermost bark layer under the measuring pin. The still largely unknown processes of bark decomposition (Gricar *et␣al*., 2015) are mainly assumed to occur during winter and therefore also have little relevance for this work.

Essential for the quality of the results is the reliability of the zero‐growth approach. The approach builds on the widely accepted turgor threshold theory for cell growth (Lockhart, 1965; Steppe *et␣al*., 2006; Muller *et␣al*., 2011; Lazzarin *et␣al*., 2019; Cabon *et␣al*., 2020; Peters *et␣al*., 2021) and is based on the assumption that the turgor threshold for growth cannot be exceeded once a tree stem starts to shrink (Zweifel *et␣al*., 2016). The approach has been shown to be largely reliable based on several indirect tests and theoretical considerations on a handful of temperate tree species, but lacks an ultimate test with an independent measurement method. Therefore, a residual uncertainty about the accuracy of the approach remains. Any deviation of the initial stem shrinkage from the stop of cell growth would affect the results presented. However, even if the time at which a tree stem begins to grow, determined by the zero‐growth approach, were not absolutely precise, this would hardly affect the growth response curves shown, since stem shrinkage occurs quite rapidly at dawn, which means that crossing the threshold for growth would only be delayed for a short period of probably less than an hour. Such a systematic shift in initial growth would not affect the daily VPD‐GRO or SWP‐GRO patterns, but could in the worst case lead to slightly shifted VPD and SWP ranges for growth. However, this reasoning does not cover the objection if someone disputes the correctness of the turgor threshold theory for growth, which is the most critical premise of this article.

### Conclusions

Hourly‐resolved growth data opened a new dimension in the analysis of stem growth responses to changing environmental conditions. Diel growth dynamics as a function of VPD and SWP showed robust general patterns as well as small but consistent, species‐specific deviations from them. The dependence of growth on very low atmospheric water demand (low VPD) and its temporal decoupling from periods of photosynthesis suggests that carbon allocation to radial stem growth is mainly sink‐driven on a diel scale. It also suggests that growth depends primarily on tree water conditions and only secondarily on current carbon allocation, at least in the short term.

The higher drought sensitivity of the growth process compared to the carbon assimilation process may explain why even starving trees do not exhaust their carbon reserves under drought stress, since in a dry period, carbon assimilation seems possible for longer than radial stem growth. The fact that the timing of peak growth within the 24‐h cycle has such strong explanatory power for the overall growth performance of a species suggests a subtly balanced interplay between the different carbon source and sink dynamics and highlights the close link between a tree's water relations and its growth. The findings that trees grow mainly at night and that VPD, in addition to SWP, has a strongly limiting influence on growth is key to better understand climate change effects on forest growth dynamics.

## Author contributions

Conceptualization: RZ, FS, SE. Methodology: RZ, FS, NB, WE, AG, MH, RLP, LW, MW, KZ, SE. Investigation: RZ, SB, MH, LW, MW, SE. Visualization: RZ, RLP, SE, KZ. Funding acquisition: RZ, NB, WE, LW, SE. Project administration: RZ, SE. Supervision: RZ, WE, SB, SE. Writing – original draft: RZ, FS, SB, NB, WE, AG, MH, RLP, LW, MW, KZ, SE.

## Supporting information

**Fig.␣S1** Scheme␣of processes determining stem radius changes (SRCs) measured by point dendrometers mounted on the stem surface, and a data example.
**Fig.␣S2** Comparison of soil water potential (SWP) data from different soil depths.
**Fig.␣S3** Species‐specific, hourly‐resolved, radial stem growth in the measured space of vapour pressure deficit (VPD) and soil water potential (SWP).
**Fig.␣S4** Comparison of measured growth data with the interpolated ones in the contour plots.
**Fig.␣S5** Growth response patterns in relation to vapour pressure deficit (VPD) and soil water potential (SWP) grouped for the months April to August.
**Fig.␣S6** Examples of stem growth under moderately dry soil conditions.
**Table␣S1** Tree species and its occurrence at the different sites with different expositions and vegetation compositions.
**Table␣S2** Species‐specific averages of vapour pressure deficit (VPD) and soil water potential (SWP) for hours with stem growth.
**Table␣S3** Explanatory power of vapour pressure deficit (VPD) and soil water potential (SWP) for diel growth with multiple linear regression models.
**Table␣S4** Explanatory power of environmental variables for hourly‐resolved diel growth with multiple linear regression models.
Please note: Wiley Blackwell are not responsible for the content or functionality of any Supporting Information supplied by the authors. Any queries (other than missing material) should be directed to the *New Phytologist* Central Office.

## Data Availability

All underlying data is made available in a data repository (https://www.pangaea.de).
