# Supplementary material for: Why trees grow at night
Source: New Phytol. 2021 Jul 7;231(6):2174–85. doi: 10.1111/nph.17552 (PMC8457160; doi:10.1111/nph.17552)
Supplement: Supplementary file 1 — Fig.␣S1 Scheme␣of processes determining stem radius changes (SRCs) measured by point dendrometers mounted on the stem surface, and a data example. Fig.␣S2 Comparison of soil water potential (SWP) data from different soil depths. Fig.␣S3 Species‐specific, hourly‐resolved, radial stem growth in the measured space of vapour pressure deficit (VPD) and soil water potential (SWP). Fig.␣S4 Comparison of measured growth data with the interpolated ones in the contour plots. Fig.␣S5 Growth response patterns in relation to vapour pressure deficit (VPD) and soil water potential (SWP) grouped for the months April to August. Fig.␣S6 Examples of stem growth under moderately dry soil conditions. Table␣S1 Tree species and its occurrence at the different sites with different expositions and vegetation compositions. Table␣S2 Species‐specific averages of vapour pressure deficit (VPD) and soil water potential (SWP) for hours with stem growth. Table␣S3 Explanatory power of vapour pressure deficit (VPD) and soil water potential (SWP) for diel growth with multiple linear regression models. Table␣S4 Explanatory power of environmental variables for hourly‐resolved diel growth with multiple linear regression models. Please note: Wiley Blackwell are not responsible for the content or functionality of any Supporting Information supplied by the authors. Any queries (other than missing material) should be directed to the New Phytologist Central Office. [file NPH-231-2174-s001.pdf]

New Phytologist

New Phytologist **Supporting Information**

Article title: **Why trees grow at night**
Authors: Roman Zweifel, Frank Sterck, Sabine Braun, Nina Buchmann, Werner Eugster, Arthur Gessler, Matthias Häni, Richard L. Peters, Lorenz Walthert, Micah Wilhelm, Kasia Ziemińska, and Sophia Etzold
Article acceptance date: 07 June 2021

The following Supporting Information is available for this article:

Fig. S1. Scheme of processes determining stem radius changes (SRC) measured by point dendrometers mounted on the stem surface, and a data example.

Fig. S2. Comparison of soil water potential (SWP) data from different soil depths.

Fig. S3. Species-specific, hourly-resolved, radial stem growth in the measured space of vapor pressure deficit (VPD) and soil water potential (SWP).

Fig. S4. Comparison of measured growth data with the interpolated ones in the contour plots.

Fig. S5. Growth response patterns in relation to vapour pressure deficit (VPD) and soil water potential (SWP) grouped for the months April to August.

Fig. S6. Examples of stem growth under moderately dry soil conditions.

Table S1. Tree species and its occurrence at the different sites with different expositions and vegetation compositions**.**

Table S2. Species-specific averages of vapor pressure deficit (VPD) and soil water potential (SWP) for hours with stem growth.

Table S3. Explanatory power of vapour pressure deficit (VPD) and soil water potential (SWP) for diel growth with multiple linear regression models.

Table S4. Explanatory power of environmental variables for hourly-resolved diel growth with multiple linear regression models.

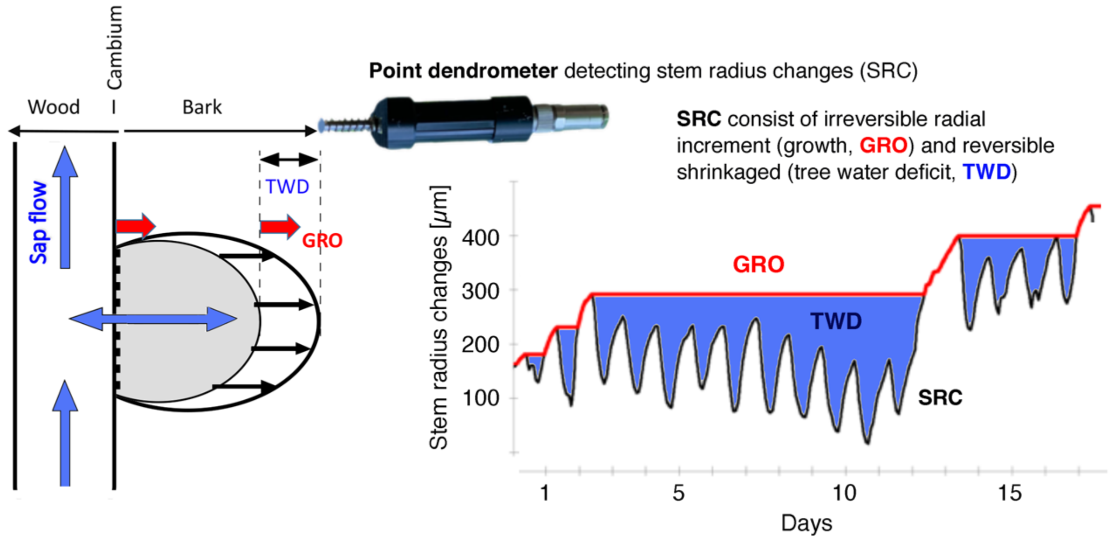

Fig. S1. Scheme of processes determining stem radius changes (SRC) measured by point dendrometers mounted on the stem surface, and a data example. New cells formed in the cambium become either xylem or phloem and induce an irreversible expansion of the stem radius, here called growth (GRO). Water-induced changes in the water potential in the stem lead to reversible shrinkage and expansion mainly of the bark. Stem shrinkage is thus the result of water withdrawal from the bark into the xylem and is therefore called tree water deficit-induced shrinkage of the stem (or tree water deficit, TWD). This process is completely reversible, as the water returns to the bark and the stem expands again when the water potential gradient changes. According to the turgor threshold theory (Lockhart, 1965) and the zero growth concept (Zweifel *et al.*, 2016), cell division and cell expansion stops almost completely when the tree trunks start to shrink. The 17-day plot shows how SRC is separated into GRO and TWD based on the underlying assumptions.

References:

Lockhart JA. 1965. An analysis of irreversible plant cell elongation. *Journal of Theoretical Biology* **8**: 264-275.

Zweifel R, Haeni M, Buchmann N, Eugster W. 2016. Are trees able to grow in periods of stem shrinkage? *New Phytologist* **211**: 839-849.

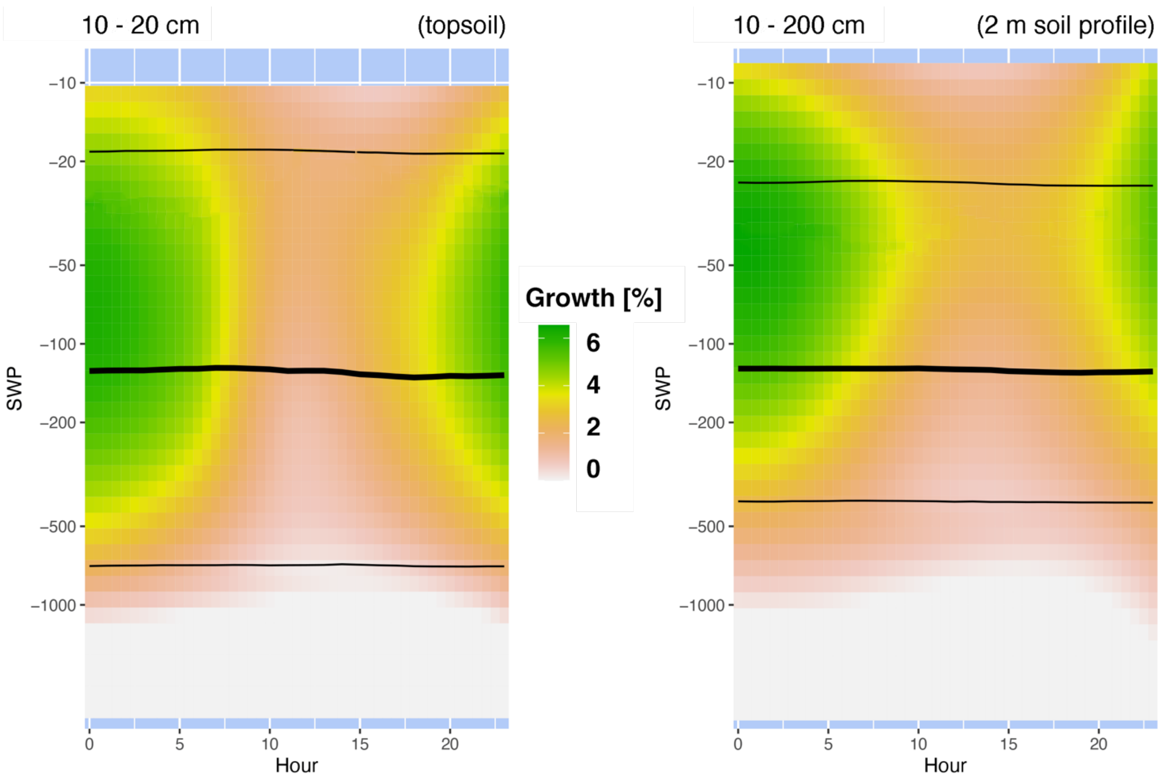

Fig. S2. Comparison of soil water potential (SWP) data from the topsoil (10-20 cm), available for the entire network, **with data from 2 m soil profiles (10-200 cm)** available at the five sites Sihlwald_1178 (beech), Saillon_1570 (beech), Chippis_1575 (pine), Chippis_1660 (pubescent oak), and Gampel (spruce). The contour plots show hourly-resolved radial stem growth over 24 hours in relation to soil water potential data from the topsoil and from 2 m soil profiles. Growth is shown as relative contribution of a specific hour to annual growth. The bold black line indicates the median SWP measured during the growth period, the thin black lines correspond to the 25- and 75% quantiles. The temporal growth response pattern largely remained the same, whereas the values on the y-axis of the topsoil profiles were 200-300 kPa higher than in the 2 m profiles. The interpolation output was restricted to the effectively measured range of SWP. Blue areas indicate no data.

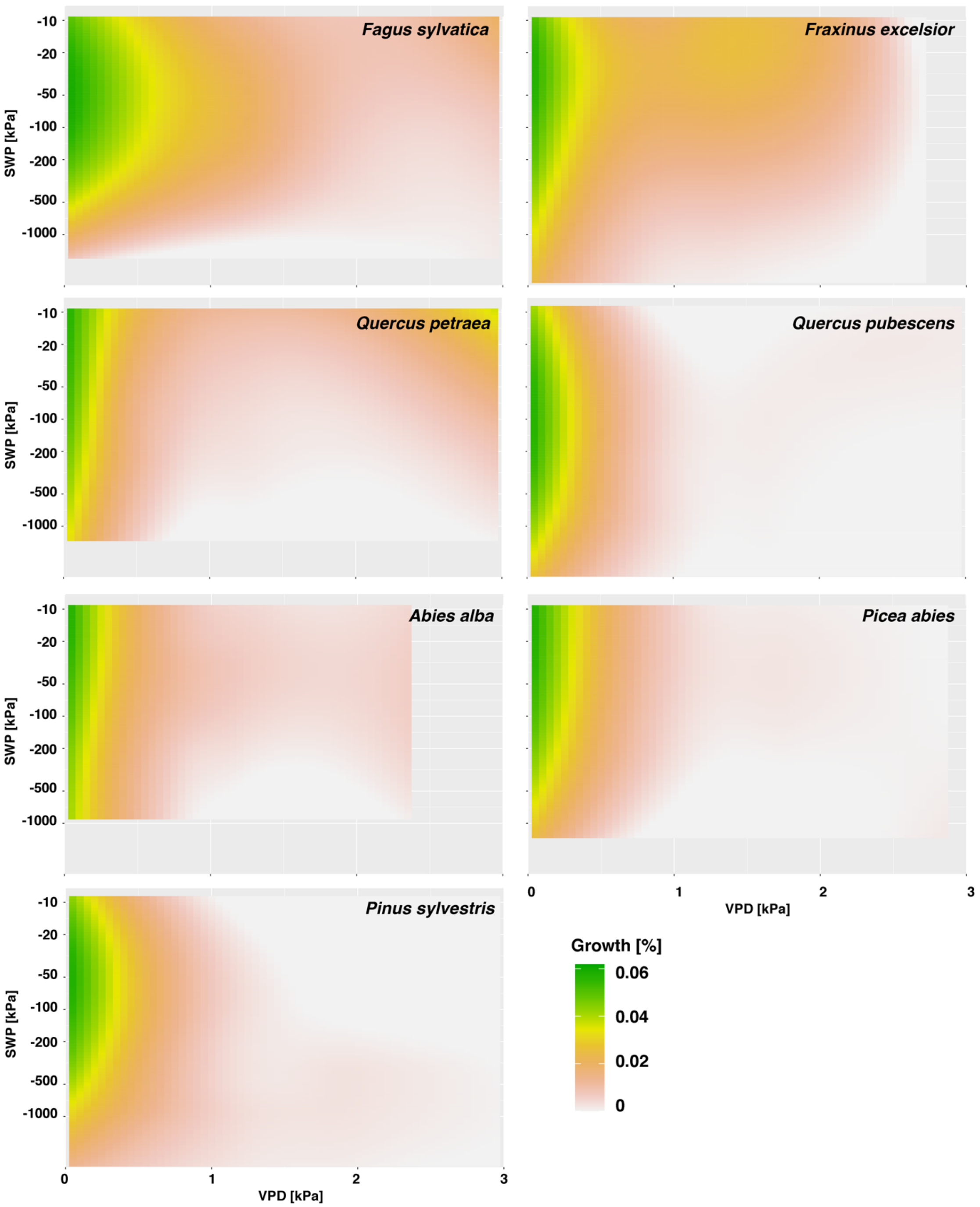

Fig. S3. Species-specific, hourly-resolved, radial stem growth in the measured space of vapor pressure deficit (VPD) and soil water potential (SWP). Growth was quantified as the relative hourly contribution to the total annual growth (per calculated grid element) and ranged from white (no growth, 0%), over red (marginal growth, 0.02%) to dark green (high growth, 0.06%). The interpolation output of the contour diagram was restricted to the effectively measured range of environmental variables. Grey areas indicate no data.

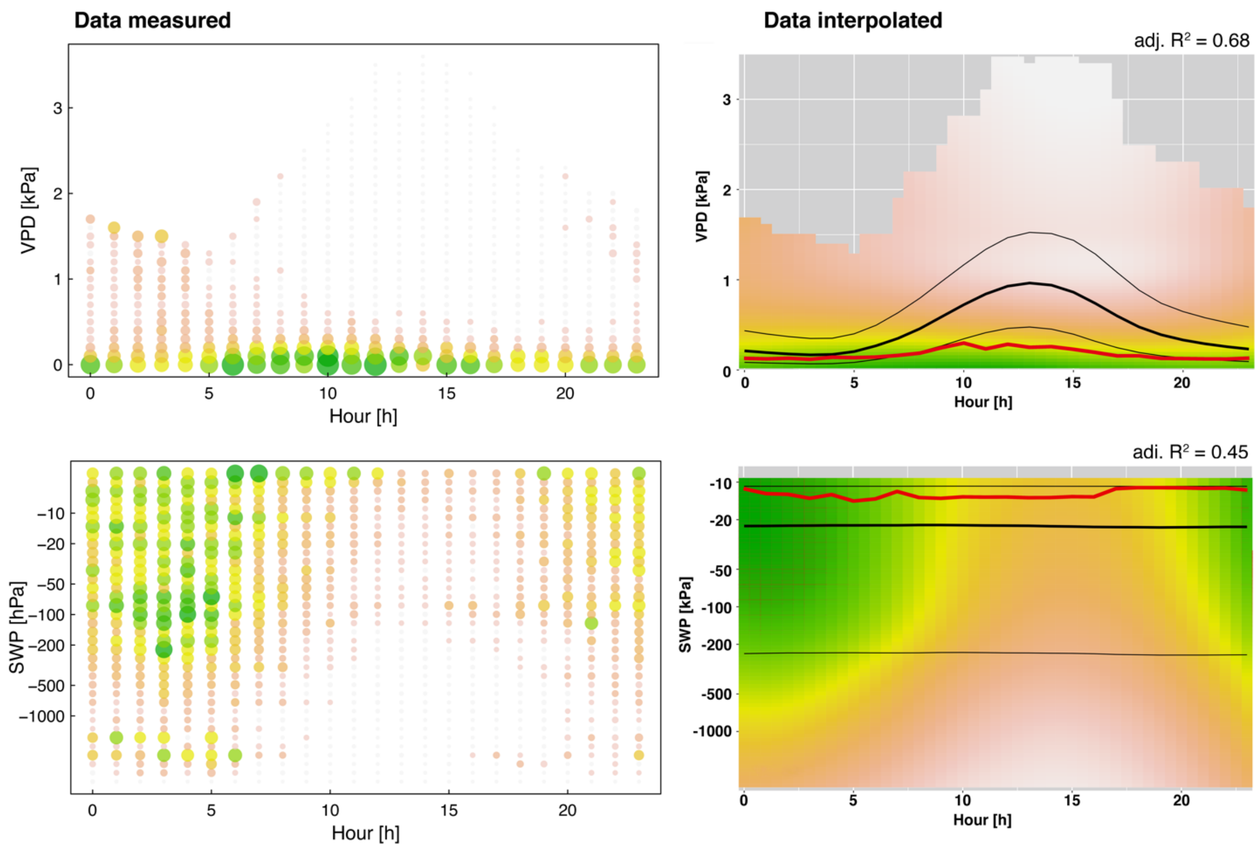

Fig. S4. Comparison of measured growth data with the interpolated ones (contour plots shown in Fig 4 of the main manuscript)**.** Growth was quantified as the relative hourly contribution to the total annual growth (per calculated grid element) and ranged from white (no growth, 0%), over red (marginal growth, 0.02%) to dark green (high growth, 0.06%). The bold black line indicates the median VPD and SWP conditions in the stem growth period (thin black lines indicate 25- and 75%-quantiles). In contrast, the bold red line shows the same but for hours with growth only. Data shown are in an hourly resolution and pooled for all species. Contour diagrams (right panels) are based on a local polynomial regression (loess) function that interpolates growth in relation to time of day and environmental conditions. Adj. R^2 give the percentage of variation the model covered.

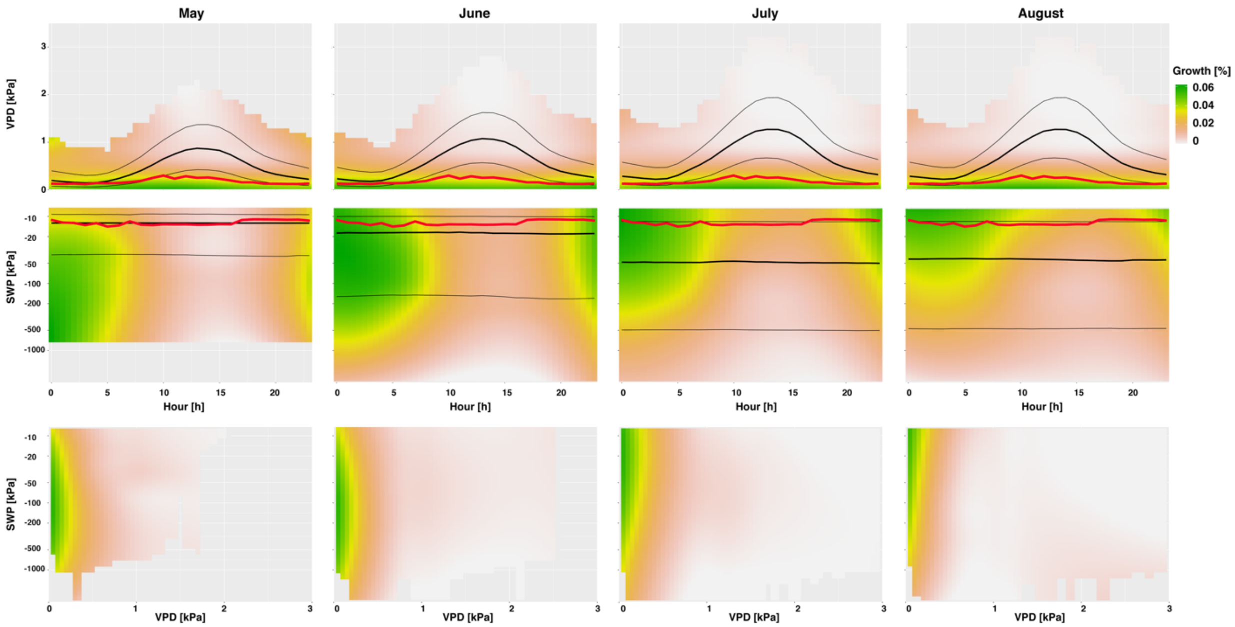

Fig. S5. Growth response patterns in relation to vapour pressure deficit (VPD) and soil water potential (SWP) grouped for the months April to August. The growth response patterns appeared to be largely independent from the time within the growth period. Growth was quantified as the relative hourly contribution to the total annual growth (per calculated grid element) and ranged from white (no growth, 0%), over red (marginal growth, 0.02%) to dark green (high growth, 0.06%). The bold black line indicates the median VPD and SWP conditions in the stem growth period (thin black lines indicate 25- and 75%-quantiles). In contrast, the bold red line shows the same but for hours with growth only. Data shown are in an hourly resolution and pooled for all species. Contour diagrams (right panels) are based on a local polynomial regression (loess) function that interpolates growth in relation to time of day and environmental conditions.

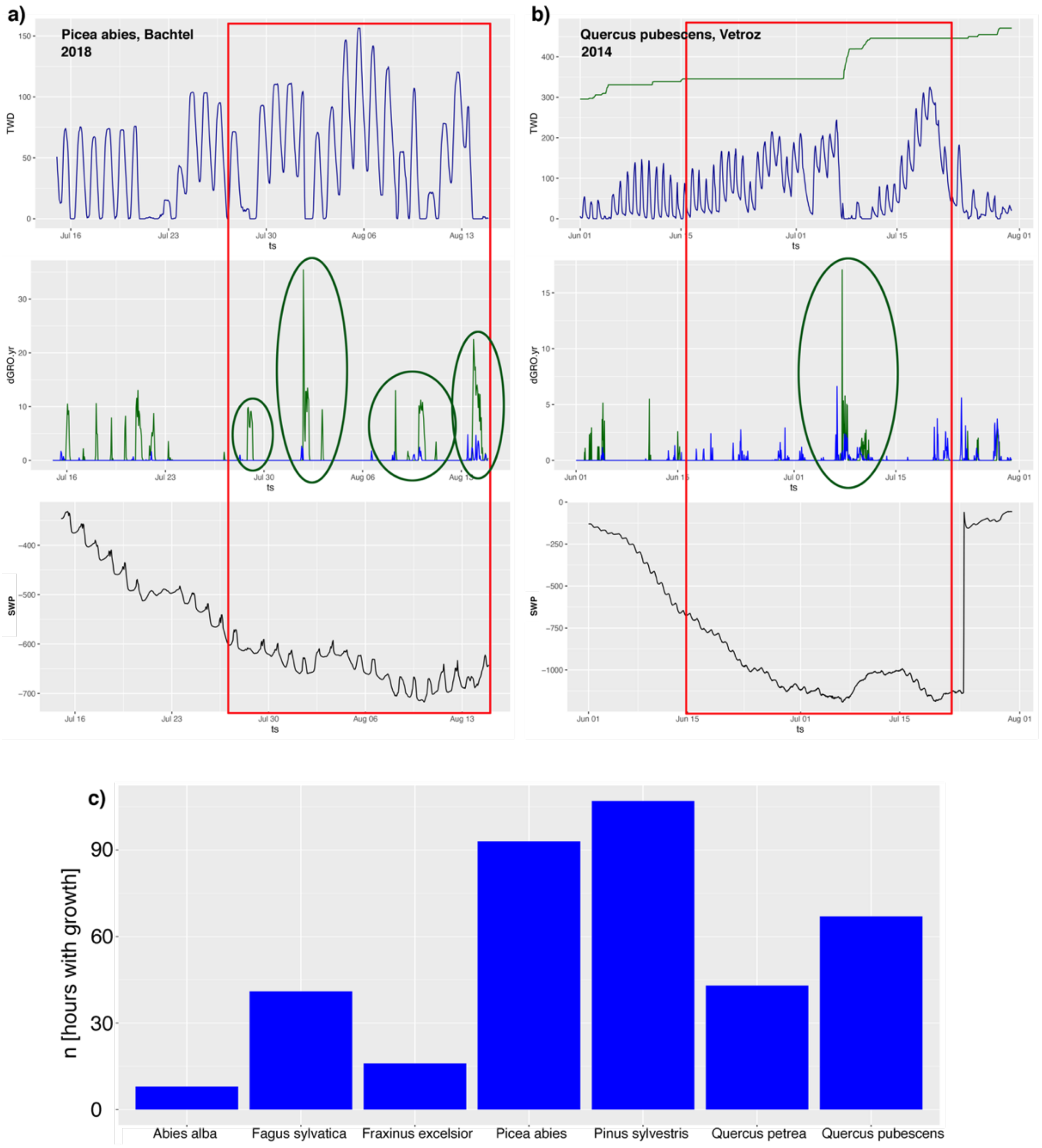

Fig. S6. Examples of stem growth under moderately dry soil conditions measured at a depth of 10-20 cm (soil water potentials <-600 kPa), **a)** *Picea abies* at Bachtel, dGRO.yr = growth rate per hour (dark green) and precipitation (blue bars). **b)** *Quercus pubescens* at Vétroz. Red frames indicate periods of low SWP, green circles show events of growth. **c)** Number of hours with growth (n, dGRO.yr > 10 µm) under dry soil conditions (SWP < -600 kPa) summed up for the entire data set and grouped by species.

Table S1. Tree species and its occurrence at the different sites with different expositions and vegetation compositions (see also Fig. 1). Sites: number of sites the species is occurring. Trees: number of trees equipped with a dendrometer. RD: underlying raw data points in a 10 min resolution (source: www.treenet.info, data set L2M_2020), ALT: range of altitudes, MAT: range of mean annual temperatures of the sites (sources: www.treenet.info, www.lwf.ch, www.meteoswiss.admin.ch), MAP: range of mean annual sums of precipitation at the sites (source: MeteoSwiss as 'Combiprecip' data combining lidar data with ground-based measurements1), DBH: range of tree stem diameters at breast height (source: www.treenet.info, metadata set L2M_2020), TH: range of tree heights (source: www.treenet.info, metadata set L2M_2020).

**Species**	**Sites [n]**	**Trees** [n]	**Forest types**	**RD** [Mio]	**ALT** [m asl]	**MAT** [°C]	**MAP** [mm]	**DBH** [cm]	**TH** [m]
Abies alba	5 (Beatenberg, Laegeren_FF, Laegeren_Hut, Schaenis_high, Vordemwald)	10	Coniferous, Mixed	3.7	480 to 1510	5.6 to 9.9	828 to 1694	34 to 88	23 to 41
Fagus sylvatica	20 (Bachtel_Beech, Birmensdorf, Chamoson_1658, Chamoson_1568, Laegeren_FF, Lausanne, Muri_Beech, Neunkirch_1134, Neunkirch_898, Riehen, Sagno_SW, Saillon_1569, Saillon_1570, Saillon_1723, Schaenis_low, Schaenis_high, Sempach, Sihlwald_1178, Vordemwald, Wangen	45	Deciduous, Mixed	13.7	410 to 880	9.0 to 11.9	549 to 1684	14 to 90	8 to 42
Fraxinus excelsior	4 Laegeren_FF, Laegeren_Hut, Schaenis_low, Visp	7	Mixed	2.6	690 to 733	9.2 to 9.9	622 to 1694	14 to 65	8 to 40
Picea abies	19 Bachtel_Spruce, Beatenberg, Birmensdorf, Davos, Grosswangen, Gampel, Laegeren_FF, Lausanne, Laegeren_Hut, Muri_Spruce, Illgraben_1724, Illgraben_1725, Sagno_SE, Schmitten, Scuol-Tarasp_1560, Surava_1558, Vordemwald, Wangen	62	Coniferous, Mixed	23.0	480 to 1650	4.5 to 11.9	663 to 1482	22 to 103	15 to 40
Pinus sylvestris	11 Alvaneu_1552, Alvaneu_1553, Chippis_1575, Lens_890, Pfynwald, Scuol-Tarasp_1560, Scuol-Tarasp_108, Surava_1122, Surava_1558, Visp, Wangen_Forest	24	Coniferous, Mixed	7.3	490 to 1520	4.8 to 10.5	622 to 945	15 to 57	8 to 25
Quercus petraea	4 Bueren_458, Jussy, Neunkirch_1657, Riehen	10	Deciduous, Mixed	3.4	460 to 630	9.3 to 11.3	834 to 1126	18 to 68	12 to 27
Quercus pubescens	6 Chamoson_1567, Chippis_1660, Saillon_1659, Saillon_1569, Vetroz_1571, Visp	12	Deciduous, Mixed	3.3	630 to 870	9.9 to 11.2	549 to 638	14 to 31	6 to 14

[1]https://www.meteoschweiz.admin.ch/content/dam/meteoswiss/de/service-und-publikationen/produkt/raeumliche-daten-combiprecip/doc/ProdDoc_CombiPrecip.pdf

Table S2. Species-specific averages of vapor pressure deficit (VPD) and soil water potential (SWP) for hours with stem growth (bold red line in Fig. 4). Listed are values covering 50%, 75% and 90% of the respective range of conditions with growth.

	VPD_{50} [kPa]	VPD_{75} [kPa]	VPD_{90} [kPa]	SWP_{50} [kPa]	SWP_{75} [kPa]	SWP_{90} [kPa]
**All species**	**0.15**	**0.37**	**0.69**	**-11**	**-39**	**-165**
A. alba	0.14	0.39	0.69	-6	-14	-47
F. sylvatica	0.24	0.53	0.88	-13	-46	-250
F. excelsior	0.19	0.56	0.9	-11	-34	-110
P. abies	0.11	0.31	0.57	-11	-39	-90
P. sylvestris	0.13	0.23	0.35	-20	-59	-311
Q. petraea	0.15	0.37	0.69	-11	-16	-165
Q. pubescens	0.17	0.35	0.62	-65	-307	-688

Table S3. Explanatory power of vapour pressure deficit (VPD) and soil water potential (SWP) for diel growth. Listed are the results of species-specific multiple linear regression models for growth with the variables VPD, SWP, and the respective interaction (VPD:SWP). adj.R^2 = explanatory power of the model. ΔR^2 = the explanatory power added by SWP. Significance of results: ns = not significant, * $p < 0.05$, ** $p < 0.01$, *** $p < 0.001$.

	**Multiple linear regression model for diel growth**						
	**VPD (regression coefficient)**		**SWP (regression coefficient)**		**VPD:SWP**	**adj.R^2**	**ΔR^2**
A. alba	-1.19	***	0.85	***	ns	0.87	0.27
F. sylvatica	-1.43	***	0.65	**	ns	0.87	0.06
F. excelsior	-1.09	***	0.42	***	ns	0.80	0.09
P. abies	-1.77	***	0.94	***	ns	0.84	0.19
P. sylvestris	-3.04	***	1.48	**	ns	0.63	0.13
Q. petraea	-1.50	***		ns	ns	0.94	0.00
Q. pubescens	-0.99	**	0.68	*	ns	0.74	0.06
**All species**						**0.81**	**+0.11**

Table S4. Explanatory power of environmental variables for hourly-resolved diel growth (relative contribution to annual growth). Listed are the results of species-specific multiple linear regression models for diel, hourly-resolved growth with the variables relative air humidity (RelH), air temperature (Temp), net radiation (Rad), soil water potential (SWP), and the respective interactions rad:swp and temp:swp. Other interactions fell out of the model. R^2 = explanatory power of the growth model. Significance of results: ns = not significant, * $p < 0.05$, ** $p < 0.01$, *** $p < 0.001$.

	**RelH**	**Temp**	**SWP**	**Rad**	**Rad:Swp**	**Temp:Swp**	**R^2**
A.alba	***	n.s.	***	n.s.	n.s.	n.s.	0.89
F.sylvatica	n.s.	n.s.	***	***	n.s.	n.s.	0.92
F.excelsior	***	n.s.	*	n.s.	n.s.	n.s.	0.83
P.abies	***	n.s.	***	n.s.	n.s.	n.s.	0.87
P.sylvestris	***	n.s.	***	n.s.	n.s.	n.s.	0.81
Q.petraea	***	n.s.	n.s.	n.s.	n.s.	n.s.	0.97
Q.pubescens	n.s.	***	n.s.	***	n.s.	n.s.	0.89
**Average**							**0.88**
